# Serum cytokine levels for predicting immune-related adverse events and the clinical response in lung cancer treated with immunotherapy

**DOI:** 10.3389/fonc.2022.923531

**Published:** 2022-08-24

**Authors:** Ni Zhao, Ye Yi, Wen Cao, Xiao Fu, Nan Mei, Chunli Li

**Affiliations:** ^1^ Department of Medical Oncology, The First Affiliated Hospital of Xi’’an Jiaotong University, Xi’an, China; ^2^ Department of Respiratory Medicine, The First Affiliated Hospital of Xi’an Jiaotong University, Xi’an, China; ^3^ Department of Hematology. The First Affiliated Hospital of Xi’an Jiaotong University, Xi’an, China

**Keywords:** biomarkers, cytokines, immunotherapy, lung cancer, immune related adverse events

## Abstract

**Background:**

At present, immunotherapy has become an important treatment for lung cancer. With the widespread use of immune checkpoint inhibitors (ICIs), we must be strict with the emergence of immune related adverse events (irAEs). There are also some patients who do not respond to immunotherapy. However, there was no biomarkers to predict the safety and efficacy of immunotherapy. The selection of immunotherapy beneficiaries contributes to improving the efficacy and safety of lung cancer treatment.

**Method:**

The electronic medical records of 221 lung cancer patients with complete clinical data who received immunotherapy from the First Affiliated Hospital of Xi ‘an Jiaotong University from November 2020 to October 2021 were collected and followed up. IBM SPSS Statistic 26.0 and R 4.1.2 software were used for statistical analysis and mapping.

**Results:**

1. A total of 221 lung cancer patients receiving immunotherapy were included in the study. Higher baseline levels of IL-1β (7.88 vs 16.16pg/mL, *P*=0.041) and IL-2 (1.28 vs 2.48pg/mL, *P*=0.001) were significantly associated with irAEs. Higher levels of IL-5 (2.64 vs 5.68pg/mL, *P*=0.013), IFN-α (1.70 vs 3.56pg/mL, *P*=0.004) and IFN-γ (6.14 vs 21.31pg/mL, *P*=0.022) after the first cycle therapy were associated with irAEs. There was no statistical significance between cytokines and irAEs after the second cycle therapy. Higher IL-5 levels in peripheral blood (9.50 vs 3.57pg/mL, *P*=0.032) were associated with the occurrence of irAEs after the third cycle therapy. 2.The efficacy of immunotherapy was assessed in 142 lung cancer patients. There was no statistical significance between baseline cytokine levels and clinical benefit. After the first cycle therapy, the level of serum cytokines had no statistical significance with the occurrence of immunotherapy clinical benefit. Lower serum levels of IL-10 (2.66 vs 1.26pg/mL, *P*=0.016) and IL-17 (8.47 vs 2.81pg/mL, P=0.015) were associated with clinical benefit after the second cycle therapy. Lower serum levels of IL-6 (10.19 vs 41.07pg/mL, *P*=0.013) and IL-8 (8.01 vs 17.22pg/mL, *P*=0.039) were associated with clinical benefit of immunotherapy after the third cycle therapy.

**Conclusion:**

1. Baseline IL-1β and IL-2 levels in peripheral blood were associated with the occurrence of irAEs in lung cancer patients. The levels of IL-5, IFN-α and IFN-γ during treatment were associated with irAEs. 2. Baseline cytokine levels in peripheral blood were not associated with immunotherapy efficacy. The levels of IL-6, IL-8, IL-10, and IL-17 levels during treatment were associated with immunotherapy efficacy.

## Introduction

Lung cancer is the leading cause of cancer-related deaths worldwide (1).Treatment with immune checkpoint inhibitors (ICIs) has led to a shift in the treatment of solid tumors, including lung cancer (2–4). Although recent clinical studies have demonstrated that programed cell death ligand-1 (PD-L1) expression on tumor cells is associated with clinical benefits in the treatment of lung cancer (3, 5), anti ICIs is also effective in some patients whose PD-L1 levels are low in their tumor tissue (2, 4). Moreover, because of the difficulty associated with obtaining tumor tissues, the identification of prognostic biomarkers in circulating blood for patient selection in pragmatic clinical settings would be of considerable value for optimizing and personalizing immunotherapy. Some reports have also suggested that the tumor mutational burden (TMB), the neoantigen burden and the presence of tissue infiltrating lymphocytes are predictive biomarkers in ICI treatment (6, 7). But the sensitivity and specificity of these biomarkers are still insufficient.

**Figure 1:**
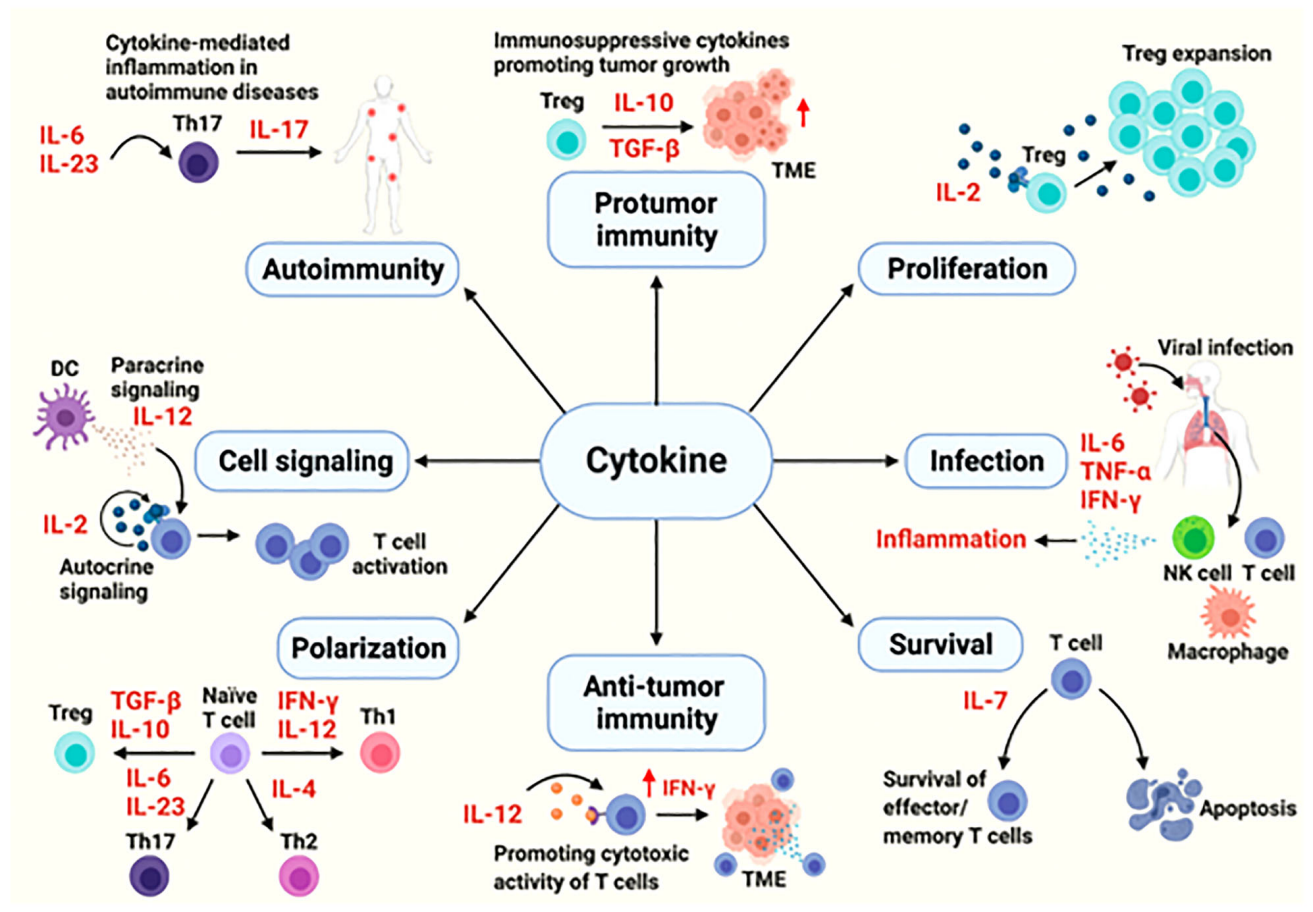
The cytokines in immune processes (8).

Cytokines are the major modulators of the innate and adaptive immune system, mainly involved in maintaining immune homeostasis and mediating immune responses related to infection, autoimmune diseases and cancer. The functions of cytokines are complex and varied. They can protect the body, and excessive activation or severe deficiency can also cause autoimmune diseases or promote the development of cancer (8) (**Figure 1**). Cytokines involved in cell communication include interleukin, IFN, some members of the TNF superfamily, chemokines and growth factors, etc. Signal transmission is mainly through paracrine and autocrine functions of these cytokines.

**Figure 2:**
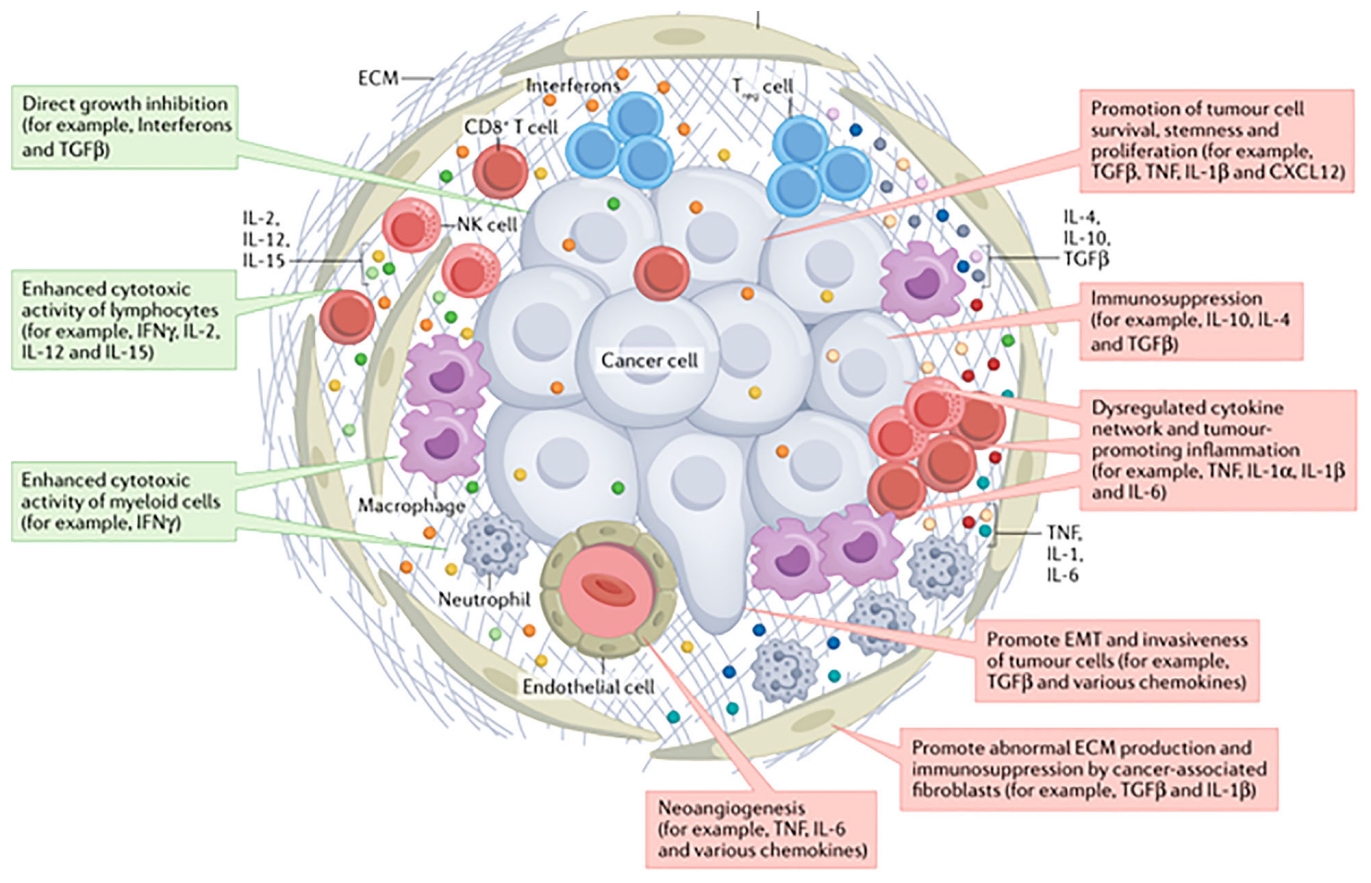
The cytokines in tumor environment (10).

An increasing number of preclinical and clinical studies have suggested that infiltrating immune cells within a tumor or the tumor cells themselves produce cytokines and chemokines, leading to modulation of the tumor microenvironment and promoting angiogenesis, growth, invasion and metastasis (9). In addition, cytokines play a functional role in promoting tumor cell growth (pro-tumor factor) or limiting tumor cell growth (anti-tumor factor) (10) (**Figure 2**). A longitudinal assessment of cytokine profiles in patients with metastatic melanoma receiving immunotherapy had reportedly established their association with irAEs progression and severe irAEs (8). Recent studies had shown that increased IL-1β and IFN-γ during treatment may be positive indicators of efficacy, while increased IL-6 during treatment might be predictive of poorer outcomes in patients with advanced NSCLC recieving immunotherapy (11).

In this study, we explored the biomarkers associated with clinical benefits such as tumor response and onset of irAEs. The aim of our study was to investigate whether a defined cytokine panel (IL-1β, IL-2, IL-4, IL-5, IL-6, IL-8, IL-10, IL-12, IL-17, IFN-α, IFN-γ, TNF-α) can play a prognostic or predictive role in lung cancer patients treated with immune checkpoint inhibitors to assess any potential correlations between their serum levels and clinical safety and the treatment response.

## Materials and methods

### Patients selection

**Figure 3:**
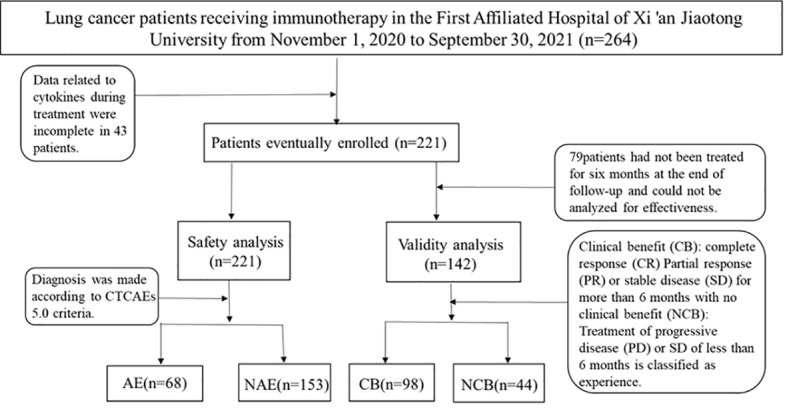
Selection process for patients.

We prospectively analyzed patients treated at the First Affiliated Hospital of Xi’an Jiaotong University from November 2020 to September 2021. Eligible patients were adults with histologically confirmed lung cancer. Patients with a previous history of systemic immunosuppressive therapy or active autoimmune disease were excluded (**Figure 3**). Agent choice was based on PD-L1 status and patients’ previous treatment history (first- or second-line setting). 221patients were selected in strict accordance with inclusion and exclusion criteria.

Toxic effects were graded with the use of the National Cancer Institute Common Terminology Criteria for Adverse Events (CTCAE version 4.0). Scheduled computed tomography or magnetic resonance imaging was performed every 9-12 weeks. Immune-related response criteria were carried out using the Response Evaluation Criteria in Solid Tumors (RECIST version 1.1).

Clinical benefit (CB) was classified as a complete response (CR), partial response (PR), or stable disease (SD) in excess of 6 months. Individuals experiencing progressive disease (PD) on therapy or who achieved SD of less than 6 months were classified as experiencing no clinical benefit (NCB).

### Cytokine testing in blood by flow cytometry

All patients collected blood samples before starting immunotherapy and the first three cycles (every 3 weeks/1 cycle, a total of four cycles), which is based on the immune system from innate response into adaptive response to determine the necessary time. Serum samples collected and processed the same standardized scheme of detecting serum cytokine IL-1β, IL-2, IL-4, IL-5, IL-6, IL-8, IL-10, IL-12, IL-17, IFN-α, IFN-γ and TNF-α levels which were worked by pairing biotin-labeled cytokines with antibodies and cytokines in the sample. Then combining with cytokine antibodies coupled with fluorescent-emitting microspheres to form sandwiches. Finally, reaction with phyoglobinin-labeled streptavidin was detected by flow cytometry. Within the detection range, fluorescence intensity was proportional to the cytokine content.

#### The sample collection and processing

Serum collection:Blood samples were collected using standard tubes. After solidification at room temperature for 30 min, centrifuged at 1000 g for 10 min.The serum was separated and sent for examination (the separated serum could be stored for 72h at -20°C).

Serum or plasma samples generally do not need to be diluted. When the detection limit is exceeded, dilute the sample according to the situation.

### Statistical analysis

The data were analyzed using IBM SPSS Statistic 26.0, and the patients were divided into two groups according to the differences between clinical characteristics, and the continuous variables were converted into dichotomous variables. For the high/low (H/L) levels of cytokines, according to the test results, those below the upper limit of the normal range were classified as the low group, and those above the upper limit were classified as the high group. Univariate analysis was conducted by χ2 test or Fisher’s exact probability. Multivariate analysis was conducted by binary logistic regression, and HR and 95% confidence interval (CI) were calculated. We analyzed the relationship between cytokine levels in peripheral blood at baseline and during treatment and the safety and efficacy of immunotherapy, the odds ratio (OR) and 95%CI results were calculated. R 4.1.2 software was used to draw a violin and nomogram to observe the distribution differences of cytokines. The independent risk factors obtained from single-factor analysis were used to construct a line graph and a predictive logistic regression model. All statistical tests were two-side probability tests (α=0.05), Throughout the analysis, *P* values less than 0.05 were considered statistically significant.

## Result

### Clinical safety

First, we evaluated baseline clinicopathological characteristics of patients that can be used to assess the safety of immunotherapy. There were significant differences in age, pathological type and PD-L1 expression status (*P* < 0.05) (as shown in **Table 1**).

**Table 1:** Relationship of clinicopathological between AE and NAE.

	AE (N=68)	NAE (N=153)	*P*
Age			**0.024**
≤64	35 (15.8%)	84 (38.0%)	
>64	33 (14.9%)	69 (31.2%)	
Sex			0.108
Male	61 (27.6%)	124 (56.1%)	
Female	7 (3.2%)	29 (13.1%)	
Smoke Status			0.579
Current or former	44 (19.9%)	93 (42.1%)	
Never	24 (10.9%)	60 (27.1%)	
Hypertension			0.186
Yes	13 (5.9%)	42 (19.0%)	
No	55 (24.9%)	111 (50.2%)	
Diabetes			0.660
Yes	5 (2.3%)	14 (6.3%)	
No	63 (28.5%)	139 (62.9%)	
Histology			**＜0.001**
NSCLC	58 (26.2%)	113 (51.1%)	
SCLC	10 (4.5%)	40 (18.1%)	
PD-L1 expression			**0.009**
Negative	13 (5.9%)	15 (6.8%)	
Positive	20 (9.0%)	27 (12.2%)	
Unknown	35 (15.8%)	111 (5.0%)	
Metastases Organ^*^			0.332
Brain metastasis	13 (5.9%)	29 (13.1%)	
Lung metastasis	18 (8.1%)	45 (20.4%)	
Liver metastasis	9 (4.1%)	34 (15.4%)	
Bone metastasis	31 (14.0%)	49 (22.2%)	
Lymph node metastasis	33 (15.0%)	79 (35.7%)	
Metastatic number			0.299
≤2	53 (24.0%)	109 (49.3%)	
>2	15 (6.8%)	44 (20.0%)	
Combined therapy			0.629
Yes	65 (29.4%)	142 (64.2%)	
No	3 (1.3%)	11 (5.0%)	
ICI treatment received			0.154
PD-1	80 (36.2%)	123 (55.7%)	
PD-L1	8 (3.6%)	30 (13.6%)	
Line of therapy			0.413
First line	44 (19.9%)	109 (49.3%)	
≥Second line	24 (10.9%)	44 (19.9%)	
DOT			0.413
≤3	19 (8.6%)	42 (19.0%)	
>3	49 (22.2%)	111 (50.2%)	

*There may be one or more distant migrations at the same time. The bold values P<0.05.

#### Relationship between baseline cytokine levels and the irAEs onset

Lung cancer patients receiving immunotherapy were divided into 2 groups according to their serum baseline cytokine levels (those not above the upper limit of the normal range were low groups). Univariate analysis showed that higher baseline IL-1β and IL-2 levels were significantly associated with the occurrence of irAEs (P ≤ 0.05). In order to exclude the influence of confounding factors, age, sex, pathological type and PD-L1 expression status were included in the regression model. The results showed that higher baseline levels of IL-1β (IL-1>12.4pg/mL) and IL-2 (IL-2>7.5pg/mL) were independent risk factors for the occurrence of irAEs. AE patients had higher baseline levels of IL-1β and IL-2 (OR=1.012, 95%CI 1.001-1.041, *P*=0.041; OR=1.743, 95% CI 1.237-2.456, *P*=0.001) (**Table 2**).

**Table 2:** Univariate and multivariate analysis results of baseline cytokine levels between AE and NAE.

Pretreatment	Univariate analysis	Multivariate analysis	
	*P*	OR (95% CI)	*P*
IL-1 (H/L)	**0.022**	**1.021 (1.001-1.041)**	**0.041**
IL-2 (H/L)	**0.029**	**1.743 (1.237-2.456)**	**0.001**
IL-4 (H/L)	–	1.052 (0.660-1.678)	0.831
IL-5 (H/L)	0.145	1.079 (0.994-1.170)	0.068
IL-6 (H/L)	0.527	1.003 (0.989-1.018)	0.658
IL-8 (H/L)	0.862	0.997 (0.977-1.017)	0.775
IL-10 (H/L)	–	1.163 (0.736-1.838)	0.517
IL-12 (H/L)	0.264	0.991 (0.961-1.021)	0.548
IL-17 (H/L)	0.512	1.031 (0.963-1.104)	0.376
IFN-α (H/L)	1.000	1.034 (0.911-1.174)	0.605
IFN-γ (H/L)	0.316	1.009 (0.992-1.026)	0.321
TNF-α (H/L)	0.167	1.081 (0.987-1.185)	0.093

“-”Indicates that a statistic cannot be computed. The bold values P < 0.05.

**Figure 4:**
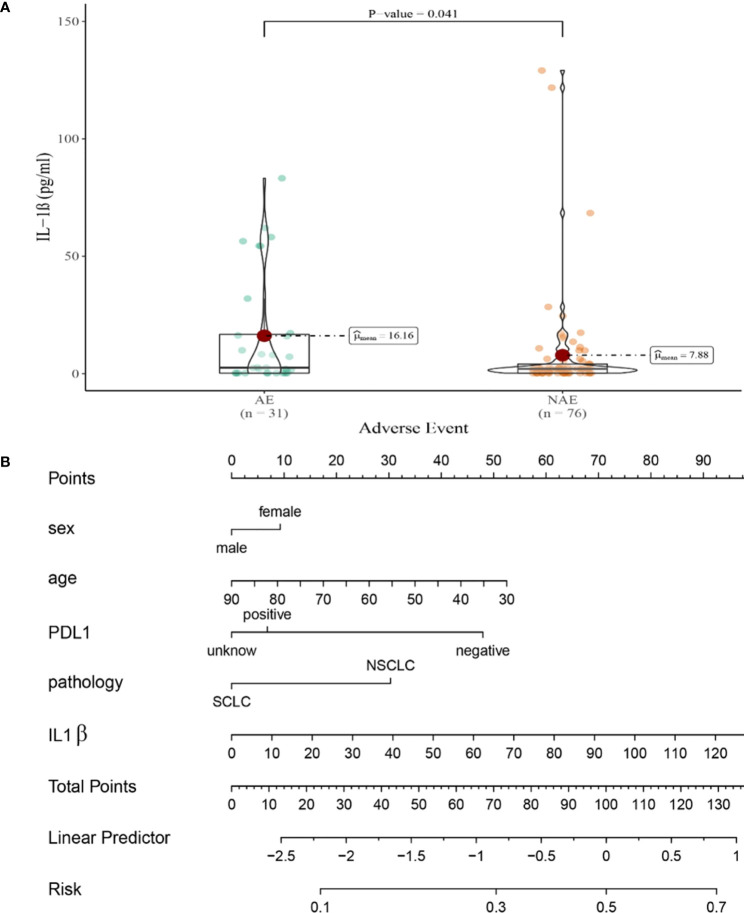
**(A)** Differences in baseline IL-1β levels between AE and NAE; **(B)** The nomogram of irAEs prediction based on logistic multivariate analysis.

Compared with NAE patients, AE patients have higher median baseline IL-1β levels (7.88 vs16.16 pg/mL, *P*=0.041, **Figure 4A**). Meanwhile, we established a nomogram based on logistic regression analysis (**Figure 4B**). As shown in the nomogram, IL-1β had a greater influence on the occurrence of AE predictions, followed by age and PD-L1 expression state, and finally gender and pathological type had less influence on the prediction of AE.

**Figure 5:**
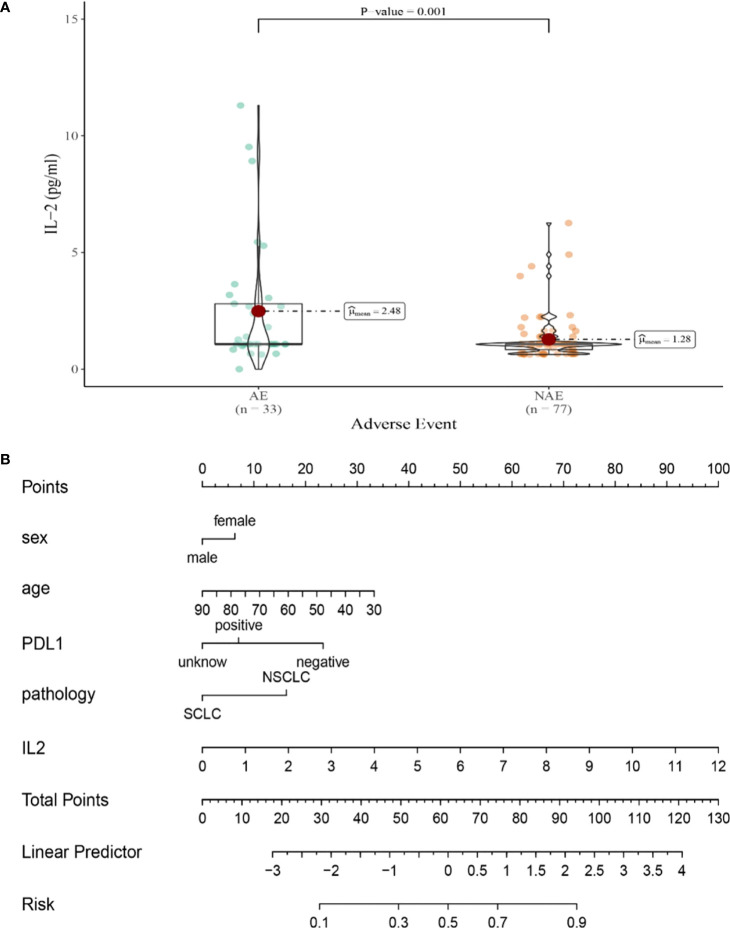
**(A)** Differences in baseline IL-2 levels between AE and NAE; **(B)** The nomogram of irAEs prediction based on logistic multivariate analysis.

Compared with NAE patients, AE patients have higher median baseline IL-2 levels (1.28 vs 2.48pg/mL, *P*=0.001, **Figure 5A**). Meanwhile, we established a nomogram based on logistic regression analysis (**Figure 5B**). As shown in the nomogram, IL-2 had a greater influence on predicting the occurrence of AE, but other factors had less influence on the prediction of AE.

#### Relationship between cytokine levels after the first cycle therapy and the irAEs onset.

Univariate analysis showed that higher IL-5 and IFN-γ levels were significantly associated with the occurrence of irAEs (*P ≤* 0.05). In order to exclude the influence of confounding factors, age, sex, pathological type and PD-L1 expression status were included in the regression model. The results showed that higher levels of IL-5 (IL-5 > 3.1pg/mL),IFN-α(IFN-α >8.5 pg/ml)and IFN-γ (IFN-γ>8.5pg/mL) after the first cycle therapy were independent risk factors for the occurrence of irAEs. AE patients from had higher levels of IL-5, IFN-α and IFN-γ after the first cycle therapy (OR=1.227, 95% CI 1.044-1.442, *P*=0.013; OR=1.055, 95% CI 1.140-1.986, P=0.004; OR=1.058, 95% CI 1.008-1.110, *P*=0.022) (**Table 3**).

**Table 3:** Univariate and multivariate analysis results of cytokine levels after the first cycle therapy between AE and NAE.

After the first cycle therapy	Univariate analysis	Multivariate analysis	
	*P*	OR (95% CI)	*P*
IL-1 (H/L)	0.742	1.022 (0.993-1.052)	0.143
IL-2 (H/L)	0.123	1.189 (0.960-1.474)	0.113
IL-4 (H/L)	1.000	0.977 (0.906-1.054)	0.549
IL-5 (H/L)	**0.008**	**1.227 (1.044-1.442)**	**0.013**
IL-6 (H/L)	0.170	0.999 (0.989-1.009)	0.848
IL-8 (H/L)	0.416	1.020 (0.976-1.065)	0.386
IL-10 (H/L)	1.000	0.994 (0.921-1.074)	0.885
IL-12 (H/L)	0.174	0.990 (0.951-1.031)	0.622
IL-17 (H/L)	0.288	1.034 (0.971-1.102)	0.294
IFN-α (H/L)	0.127	**1.505 (1.140-1.986)**	**0.004**
IFN-γ (H/L)	**0.014**	**1.058 (1.008-1.110)**	**0.022**
TNF-α (H/L)	0.282	1.115 (0.983-1.265)	0.091

“-”Indicates that a statistic cannot be computed. The bold values P < 0.05.

**Figure 6:**
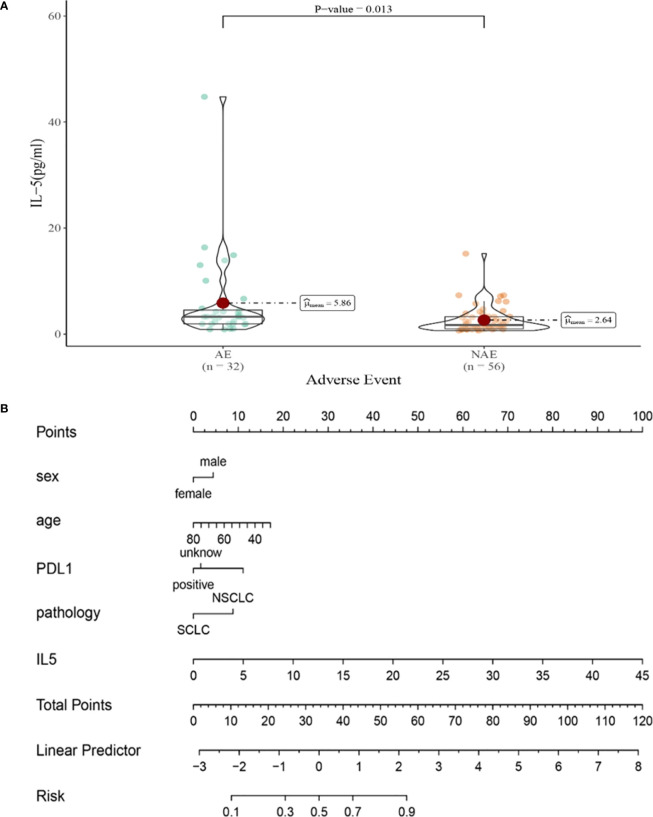
**(A)** Differences in IL-5 levels after the first cycle therapy between AE and NAE; **(B)** The nomogram of irAEs prediction based on logistic multivariate analysis.

Compared with NAE patients, AE patients have higher median IL-5 levels after the first cycle therapy (2.64 vs 5.68pg/mL, *P*=0.013, **Figure 6A**). Meanwhile, we established a nomogram based on logistic regression analysis (**Figure 6B**). As shown in the nomogram, IL-5 had a greater influence on predicting the occurrence of AE, but other factors had less influence on the prediction of AE.

**Figure 7:**
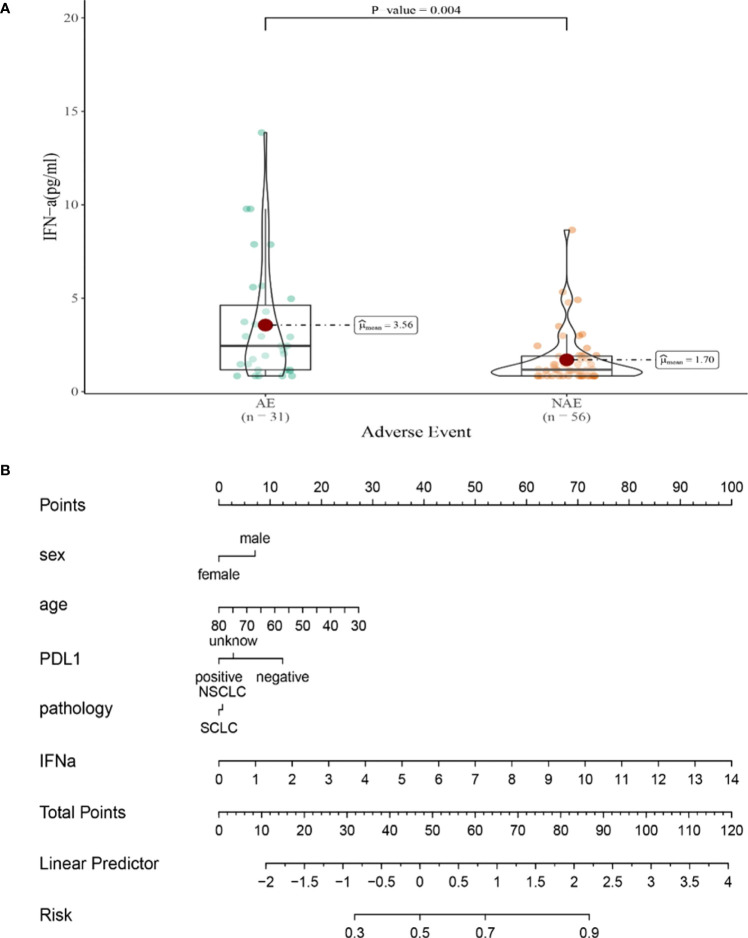
**(A)** Differences in IFN-αlevels after the first cycle therapy between AE and NAE; **(B)** The nomogram of irAEs prediction based on logistic multivariate analysis.

Compared with NAE patients, AE patients have higher median IFN-α levels after the first cycle therapy (1.70 vs3.56 pg/mL, *P*=0.004, **Figure7A**). Meanwhile, we established a nomogram based on logistic regression analysis (**Figure 7B**). As shown in the nomogram, IFN-α had a greater influence on predicting the occurrence of AE, but other factors had less influence on the prediction of AE.

**Figure 8:**
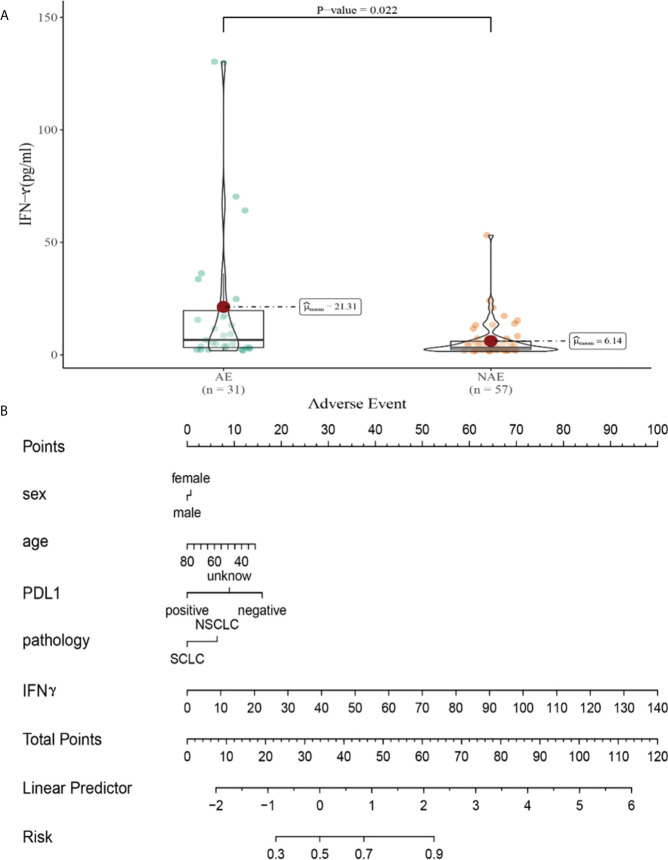
**(A)** Differences in IFN-γ levels after the first cycle therapy between AE and NAE; **(B)** The nomogram of irAEs prediction based on logistic multivariate analysis.

Compared with NAE patients, AE patients have higher median IFN-γ levels after the first cycle therapy (6.14 vs 21.31pg/m, *P*=0.022, **Figure 8A**). Meanwhile, we established a nomogram based on logistic regression analysis (**Figure 8B**). As shown in the nomogram, IFN-γ had a greater influence on predicting the occurrence of AE, but other factors had less influence on the prediction of AE.

#### Relationship between cytokine levels after the second cycle therapy and the irAEs onset.

Univariate analysis showed that higher IL-5 and IL-12 levels were significantly associated with the occurrence of irAEs (P ≤ 0.05). In order to exclude the influence of confounding factors, age, sex, pathological type and PD-L1 expression status were included in the regression model. The results showed that levels of cytokines weren’t connected with occurrence of irAEs (**Table 4**).

**Table 4:** Univariate and multivariate analysis results of cytokine levels after the second cycle therapy between AE and NAE.

After the second cycle therapy	Univariate analysis	Multivariate analysis	
	*P*	OR (95% CI)	*P*
IL-1 (H/L)	0.237	1.019 (0.999-1.039)	0.065
IL-2 (H/L)	0.345	1.043 (0.899-1.211)	0.576
IL-4 (H/L)	0.359	1.864 (0.686-5.066)	0.222
IL-5 (H/L)	**0.043**	1.089 (0.994-1.192)	0.066
IL-6 (H/L)	0.076	1.009 (0.987-1.031)	0.416
IL-8 (H/L)	0.900	1.005 (0.967-1.045)	0.801
IL-10 (H/L)	–	1.408 (0.970-2.042)	0.072
IL-12 (H/L)	**0.020**	1.482 (0.960-2.286)	0.076
IL-17 (H/L)	1.000	1.052 (0.938-1.180)	0.389
IFN-α (H/L)	0.128	1.158 (0.971-1.382)	0.103
IFN-γ (H/L)	0.622	1.017 (0.999-1.036)	0.060
TNF-α (H/L)	0.045	1.096 (0.968-1.240)	0.147

“-”Indicates that a statistic cannot be computed.

3.1.4 Relationship between cytokine levels after the third cycle therapy and the irAEs onset.

Univariate analysis showed that levels of cytokines weren’t connected with occurrence of irAEs. Multivariate analysis showed that high levels of IL-5 (IL-5 > 3.1pg/mL) after the third cycle therapy was independent risk factor for the occurrence of irAEs. Patients with AEs from immunotherapy had higher IL-5 levels (OR=1.187, 95% CI 1.015-1.388, *P* =0.032) (**Table 5**).

**Table 5:** Univariate and multivariate analysis results of cytokine levels after the third cycle therapy between AE and NAE.

After the third cycle therapy	Univariate analysis	Multivariate analysis	
	*P*	OR (95% CI)	*P*
IL-1 (H/L)	0.413	1.013 (0.991-1.035)	0.252
IL-2 (H/L)	0.298	1.068 (0.871-1.310)	0.527
IL-4 (H/L)	0.418	1.696 (0.718-4.006)	0.229
IL-5 (H/L)	0.268	**1.187 (1.015-1.388)**	**0.032**
IL-6 (H/L)	0.350	1.013 (0.988-1.038)	0.318
IL-8 (H/L)	1.000	1.016 (0.975-1.060)	0.447
IL-10 (H/L)	–	1.512 (0.994-2.298)	0.053
IL-12 (H/L)	0.425	1.100 (0.892-1.357)	0.372
IL-17 (H/L)	0.418	1.143 (0.946-1.387)	0.174
IFN-α (H/L)	0.161	1.210 (0.914-1.603)	0.183
IFN-γ (H/L)	0.113	1.013 (0.990-1.037)	0.279
TNF-α (H/L)	1.000	1.035 (0.956-1.111)	0.334

“-”Indicates that a statistic cannot be computed.

**Figure 9:**
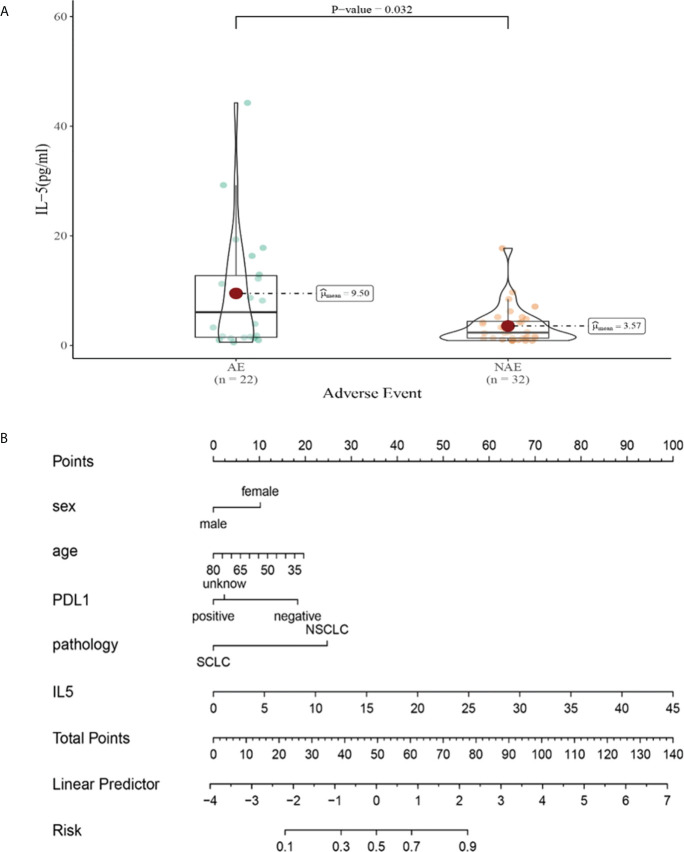
**(A)** Differences in IL-5 levels after the third cycle therapy between AE and NAE; **(B)** The nomogram of irAEs prediction based on logistic multivariate analysis.

Compared with NAE patients, AE patients have higher median IL-5 levels after the third cycle therapy (9.50 vs 3.57pg/mL, *P* = 0.032, **Figure 9A**). Meanwhile, we established a nomogram based on logistic regression analysis (**Figure 9B**). As shown in the nomogram, IL-5 had a greater influence on predicting the occurrence of AE, but other factors had less influence on the prediction of AE.

### Clinical response efficacy

We evaluated baseline clinicopathological characteristics of 142 patients that can be used to assess the clinical response efficacy of immunotherapy. There were significant differences in PD-L1 expression status and DOT (*P* < 0.05) (as shown in **Table 6**).

**Table 6:** Relationship between clinicopathological characteristics and clinical response efficacy.

	CB(N=98)	NCB(N=44)	*P*
Age			0.802
≤63	53 (37.3%)	27 (19.0%)	
>63	45 (31.7%)	17 (12.0%)	
Sex			0.950
Male	82 (57.7%)	37 (26.1%)	
Female	16 (11.3%)	7 (4.9%)	
Smoke Status			0.477
Current or former	64 (45.1%)	26 (18.3%)	
Never	3 (2.1%)	18 (12.6%)	
Hypertension			0.405
Yes	24 (16.9%)	8 (5.6%)	
No	74 (52.1%)	36 (25.4%)	
Diabetes			0.660
Yes	9 (6.3%)	4 (2.8%)	
No	89 (62.7%)	40 (28.2%)	
Histology			0.790
NSCLC	76 (53.5%)	35 (24.6%)	
SCLC	22 (15.5%)	9 (6.3%)	
PD-L1 expression			**0.050**
Negative	18 (12.7%)	2 (1.4%)	
Positive	16 (11.2%)	12 (8.5%)	
Unknown	64 (45.1%)	30 (2.1%)	
Metastases Organ^*^			0.859
Brain metastasis	17 (12.0%)	9 (6.3%)	
Lung metastasis	29 (20.4%)	13 (9.2%)	
Liver metastasis	21 (14.8%)	8 (5.6%)	
Bone metastasis	41 (28.9%)	22 (15.5%)	
Lymph node metastasis	54 (38.0%)	20 (14.1%)	
Metastatic number			0.778
≤2	69 (48.6%)	32 (22.5%)	
>2	29 (20.4%)	12 (8.5%)	
Combined therapy			0.724
Yes	90 (63.4%)	42 (29.6%)	
No	8 (5.6%)	2 (1.4%)	
ICI treatment received			0.785
PD-1	82 (57.7%)	36 (25.4%)	
PD-L1	16 (11.3%)	8 (5.6%)	
Line of therapy			0.495
First line	61 (43.0%)	30 (21.1%)	
≥Second line	37 (26.1%)	14 (9.9%)	
DOT			**＜0.001**
≤3	11 (7.7%)	15 (10.6%)	
>3	87 (61.3%)	29 (20.4%)	

*There may be one or more distant migrations at the same time.

#### Relationship between baseline cytokine levels and clinical response efficacy of immunotherapy.

Univariate and multivariate analysis showed that the baseline levels of cytokines weren’t connected with occurrence of clinical response efficacy (**Table 7**).

**Table 7:** Univariate and multivariate analysis results of baseline cytokine levels between CB and NCB.

Pretreatment	Univariate analysis	Multivariate analysis	
	*P*	OR (95% CI)	*P*
IL-1 (H/L)	0.697	1.001 (0.975-1.028)	0.937
IL-2 (H/L)	–	1.157 (0.803-1.695)	0.418
IL-4 (H/L)	–	0.365 (0.124-1.074)	0.067
IL-5 (H/L)	0.465	0.966 (0.860-1.084)	0.557
IL-6 (H/L)	0.355	0.977 (0.975-1.019)	0.766
IL-8 (H/L)	0.697	0.989 (0.963-1.015)	0.391
IL-10 (H/L)	–	0.801 (0.378-1.698)	0.562
IL-12 (H/L)	0.553	0.684 (0.402-1.164)	0.162
IL-17 (H/L)	1.000	1.066 (0.934-1.217)	0.343
IFN-α (H/L)	–	0.866 (0.599-1.252)	0.444
IFN-γ (H/L)	0.741	0.995 (0.968-1.023)	0.729
TNF-α (H/L)	0.512	1.056 (0.916-1.217)	0.451

“-”Indicates that a statistic cannot be computed.

#### Relationship between cytokine levels after the first cycle therapy and clinical response efficacy of immunotherapy.

Univariate analysis showed that higher IL-6 was significantly associated with clinical response efficacy (*P ≤* 0.05). In order to exclude the influence of confounding factors, PD-L1 expression status and duration of treatment (DOT) were included in the regression model. The results showed that levels of cytokines after the first cycle therapy weren’t connected with occurrence of clinical response efficacy (**Table 8**).

**Table 8:** Univariate and multivariate analysis results of cytokine levels after the first cycle therapy between CB and NCB.

After the first therapy	Univariate analysis	Multivariate analysis	
	*P*	OR (95% CI)	*P*
IL-1 (H/L)	1.000	0.979 (0.901-1.063)	0.611
IL-2 (H/L)	1.000	1.147 (0.547-2.407)	0.716
IL-4 (H/L)	0.326	0.957 (0.761-1.203)	0.707
IL-5 (H/L)	0.927	1.160 (0.899-1.497)	0.254
IL-6 (H/L)	**0.047**	0.989 (0.966-1.013)	0.353
IL-8 (H/L)	1.000	1.031 (0.914-1.164)	0.619
IL-10 (H/L)	0.318	0.847 (0.652-1.101)	0.215
IL-12 (H/L)	0.539	1.539 (0.489-4.845)	0.462
IL-17 (H/L)	1.000	1.135 (0.760-1.694)	0.536
IFN-α (H/L)	–	1.012 (0.564-1.817)	0.969
IFN-γ (H/L)	1.000	0.984 (0.952-1.016)	0.325
TNF-α (H/L)	–	0.969 (0.617-1.522)	0.892

“-”Indicates that a statistic cannot be computed.

#### Relationship between cytokine levels after the second cycle therapy and clinical response efficacy of immunotherapy.

Univariate analysis showed that lower IFN-α level was significantly associated with clinical benefit (*P ≤* 0.05). In order to exclude the influence of confounding factors, PD-L1 expression status and DOT were included in the regression model. The results showed that lower levels of IL-10 (IL-10<12.9pg/ml),IL-17(IL-17<21.4pg/ml) after the second cycle therapy were independent risk factors for the clinical benefit. Patients with CB from immunotherapy had lower levels of IL-10 and IL-17 after the second cycle therapy (OR=0.402, 95% CI 0.191-0.848, *P*=0.016; OR=0.776, 95% CI 0.633-0.951, *P*=0.015) (**Table 9**).

**Table 9:** Univariate and multivariate analysis results of cytokine levels after the second cycle therapy between CB and NCB.

After the second cycle therapy	Univariate analysis	Multivariate analysis	
		*P*	OR (95% CI)	*P*
IL-1(H/L)	0.108	0.966(0.933-1.001)	0.054
IL-2(H/L)	0.096	0.917(0.757-1.110)	0.374
IL-4(H/L)	1.000	0.247(0.049-1.237)	0.089
IL-5(H/L)	1.000	0.894(0.784-1.019)	0.093
IL-6(H/L)	0.221	0.977(0.935-1.021)	0.298
IL-8(H/L)	1.000	0.981(0.933-1.031)	0.447
IL-10(H/L)	–	**0.402(0.191-0.848)**	**0.016**
IL-12(H/L)	0.172	0.544(0.255-1.161)	0.115
IL-17(H/L)	0.276	**0.776(0.633-0.951)**	**0.015**
IFN-α(H/L)	**0.036**	0.675(0.439-1.038)	0.074
IFN-γ(H/L)	0.257	0.964(0.922-1.007)	0.102
TNF-α(H/L)	0.276	0.805(0.605-1.072)	0.137

“-”Indicates that a statistic cannot be computed.

**Figure 10:**
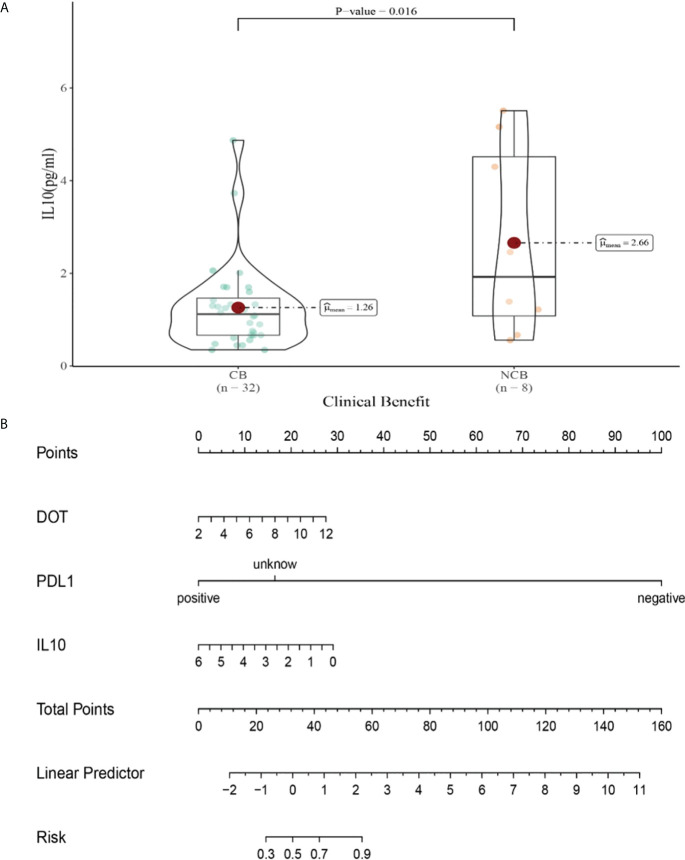
**(A)** Differences in IL-10 levels after the second cycle therapy between CB and UCB; **(B)** The nomogram based on logistic multivariate analysis.

Compared with NCB patients, CB patients have lower median IL-10 levels after the second cycle therapy (2.66 vs 1.26pg/mL, *P* =0.015, **Figure 10A**). Meanwhile, we established a nomogram based on logistic regression analysis (**Figure 10B**). As shown in the nomogram, PD-L1 had a greater influence on predicting the clinical response efficacy, but DOT and IL-10 had less influence on the prediction of CB.

**Figure 11:**
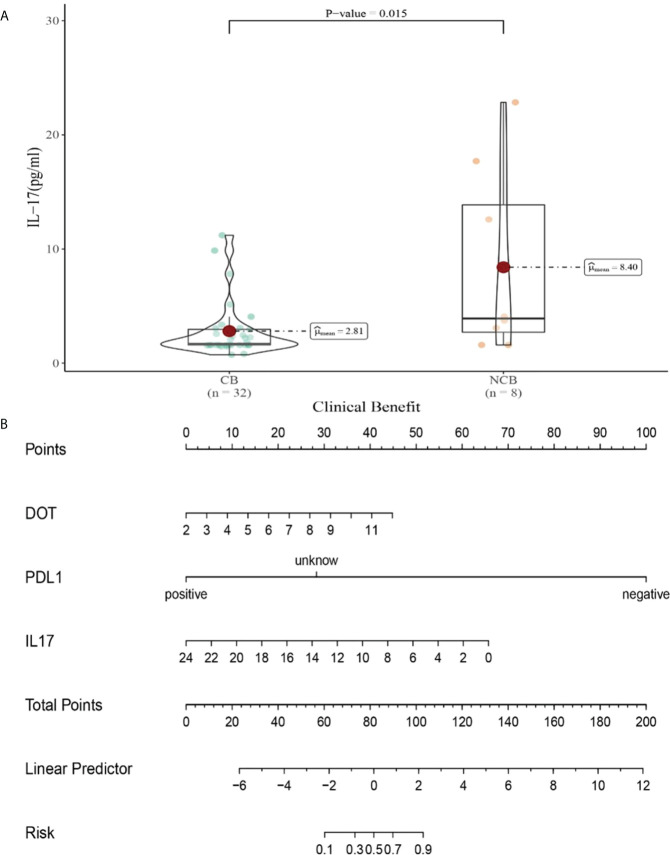
**(A)** Differences in IL-17 levels after the second cycle therapy between CB and UCB; **(B)** The nomogram based on logistic multivariate analysis.

Compared with NCB patients, CB patients have lower median IL-17 levels after the second cycle therapy (8.47 vs 2.81pg/mL, *P*=0.015, **Figure 11A**). Meanwhile, we established a nomogram based on logistic regression analysis (**Figure 11B**). As shown in the nomogram, PD-L1 and IL-17 had a greater influence on predicting the clinical response efficacy, but DOT had less influence on the prediction of CB.

#### Relationship between cytokine levels after the third cycle therapy and clinical response efficacy of immunotherapy.

Univariate analysis showed that higher IFN-α level was significantly associated with clinical benefit (*P ≤* 0.05). In order to exclude the influence of confounding factors, PD-L1 expression status and DOT were included in the regression model. The results showed that lower levels of IL-6 (IL-6<5.4pg/ml),IL-8(IL-8<20.6 pg/ml)after the third cycle therapy were independent risk factors for the clinical benefit. CB patients had lower levels of IL-6 and IL-8 after the third cycle therapy (OR=0.402, 95% CI 0.191-0.848, *P*=0.016; OR=0.776, 95% CI 0.633-0.951, *P*=0.015) (**Table 10**).

**Table 10:** Univariate and multivariate analysis results of cytokine levels after the third cycle therapy between CB and NCB.

After the third cycle therapy	Univariate analysis	Multivariate analysis	
	*P*	OR (95% CI)	*P*
IL-1 (H/L)	0.601	0.979 (0.952-1.077)	0.144
IL-2 (H/L)	0.347	0.755 (0.544-1.047)	0.092
IL-4 (H/L)	1.000	0.871 (0.432-1.758)	0.700
IL-5 (H/L)	1.000	0.929 (0.811-1.064)	0.288
IL-6 (H/L)	**0.009**	**0.936 (0.888-0.986)**	**0.013**
IL-8 (H/L)	0.163	**0.919 (0.849-0.996)**	**0.039**
IL-10 (H/L)	–	0.800 (0.486-1.315)	0.379
IL-12 (H/L)	0.573	0.711 (0.490-1.033)	0.074
IL-17 (H/L)	0.189	0.862 (0.715-1.040)	0.120
IFN-α (H/L)	0.194	0.807 (0.563-1.155)	0.240
IFN-γ (H/L)	0.073	0.970 (0.932-1.010)	0.136
TNF-α (H/L)	0.086	0.892 (0.774-1.028)	0.113

“-”Indicates that a statistic cannot be computed.

**Figure 12:**
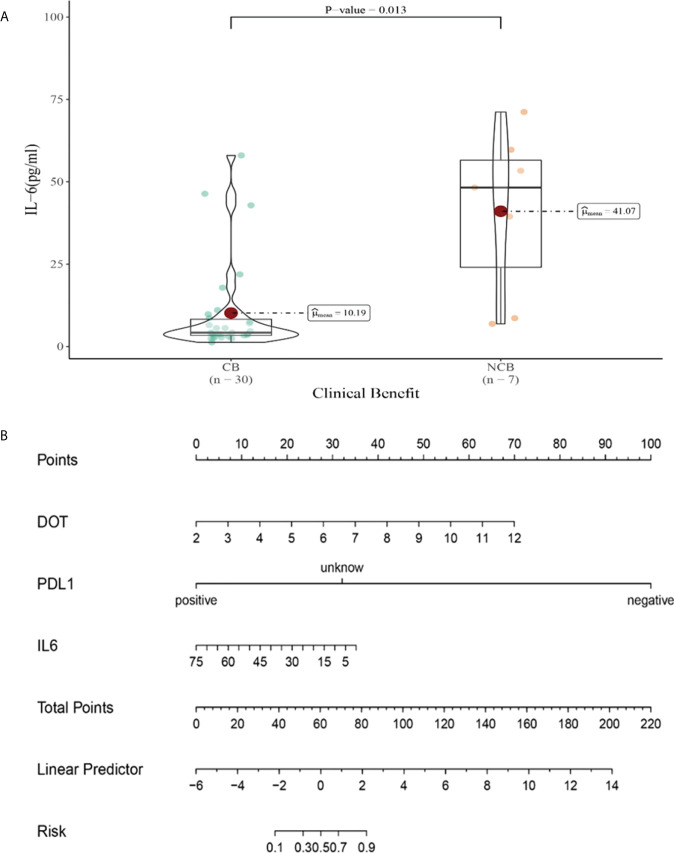
**(A)** Differences in IL-6 levels after the third cycle therapy between CB and UCB; **(B)** The nomogram based on logistic multivariate analysis.

Compared with NCB patients, CB patients have lower median IL-6 levels after the third cycle therapy (10.19 vs 41.07pg/mL, *P*=0.013, **Figure 12A**). Meanwhile, we established a nomogram based on logistic regression analysis (**Figure 12B**). As shown in the nomogram, PD-L1 and DOT had a greater influence on predicting the clinical response efficacy, but IL-6 had less influence on the prediction of CB.

**Figure 13:**
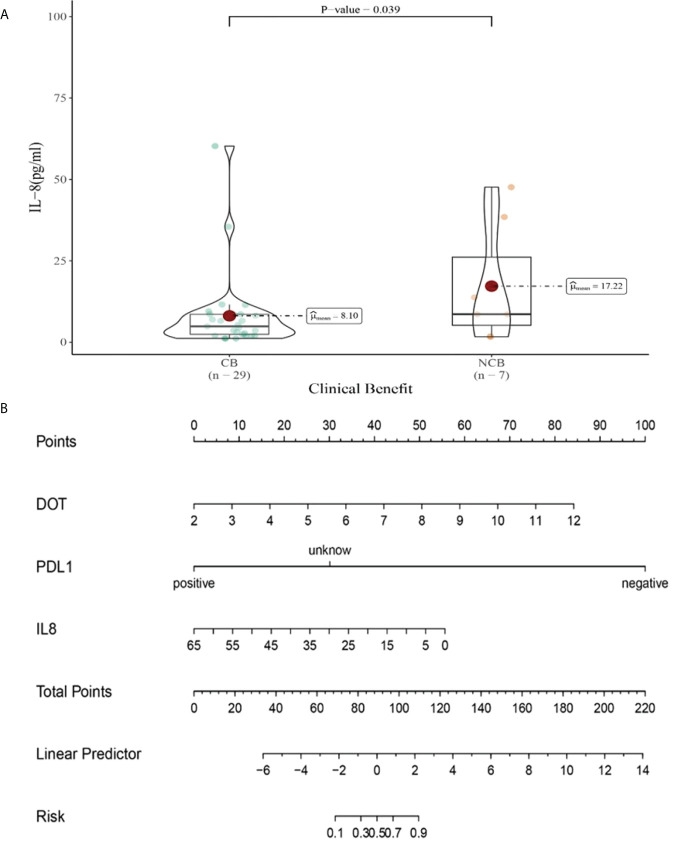
**(A)** Differences in IL-8 levels after the third cycle therapy between CB and UCB; **(B)** The nomogram based on logistic multivariate analysis.

Compared with NCB patients, CB patients have lower median IL-8 levels after the third cycle therapy (8.01 vs 17.22pg/mL, *P*=0.039, **Figure 13A**). Meanwhile, we established a nomogram based on logistic regression analysis (**Figure 13B**). As shown in the nomogram, PD-L1 and DOT had a greater influence on predicting the clinical response efficacy, but IL-8 had less influence on the prediction of CB.

## Discussion

This is the first retrospective study involving analyses of baseline and on-treatment cytokine concentrations during ICI therapy. We found that baseline levels of IL-1β and IL-2, as well as on-treatment levels of IL-5, IFN-α and IFN-γ were associated with immune-related adverse events. At the same time, on-treatment levels of IL-6, IL-8, IL-10 and IL-17 were related to the clinical response.

IL-1β is a member of the IL-1 family. After IL-1β activates IL-1, it participates in the related immune inflammatory response of lung cancer by activating NF-κB and other pathways (12). Baseline serum cytokine concentrations of IL-1β, IL-2, and GM-CSF were elevated in patients with thyroid related adverse reactions in a study of multiple solid tumors receiving immunotherapy (13). Therefore, higher baseline IL-1β levels are associated with higher levels of pro-inflammatory cytokines. If the use of immune checkpoint inhibitors at this time to activate the body’s immune cells, thereby releasing more inflammatory factors, can induce the occurrence of autoimmune response and tissue and organ damage.

Growing evidence indicates that immune-related adverse events can be tied to specific cytokines that can amplify both pro- and anti-inflammatory immunity (8). Among Th2 cytokines, IL-2 is a key cytokine involved in promoting the proliferation of natural killercells and T lymphocytes (14). Constantini (15) showed that a low serum IL-2 concentration measured at nivolumab initiation was associated with grade 3–4 toxicities in patients with advanced NSCLC.

IL-5 is mainly produced by T helper-2 (Th2) lymphocytes and Group 2 innate lymphoid cells. It can increase antibody secretion by promoting the differentiation and growth of B cells and enhance the humoral immune response mediated by Th2 cells. Immunity to tumors is mainly governed by Th1-mediated cellular immunity. A Th1-Th2 drift will lead to immunosuppression and cancer development (16).Therefore, when IL-5 levels are high during immunotherapy, the differentiation and growth of B cells are correspondingly promoted, thus increasing the secretion of antibodies, leading to the over activated humoral immune response which may attack normal tissues and organs of the body.

In cytokine analysis during immunotherapy, we observed a negative correlation between IL-6 concentration and clinical benefit in lung cancer patients after the third cycle of immunotherapy. One of the key signaling pathways controlling this phenomenon is the IL-6/JAK/STAT3 axis, which enhances tumor proliferation and cell metabolism by upregulating this signaling pathway (17, 18). Higher IL-6 levels during treatment may be indicative of high tumor cell proliferation and enhanced angiogenesis, and immunotherapy will be less effective in eliminating this state.

Another significant negative correlation with CB found in our study was the concentration of IL-8 in lung cancer patients after the third cycle of immunotherapy. Il-8 is a member of the neutrophil chemokine family (19). Studies have shown that early decreased peripheral blood IL-8 levels are associated with longer overall survival in patients with melanoma (*P*=0.001) and non-small cell lung cancer (*P*=0.015) (20). However, further analysis of peripheral blood IL-8 levels in combination with other inflammatory indicators is needed to clearly distinguish between elevated IL-8 caused by cancer progression and elevated IL-8 caused by inflammation.

At the same time, we also find that IL-10 concentration was negatively associated with CB in lung cancer patients after second cycle of immunotherapy. IL-10 is a cytokine that has both anti-inflammatory and pro-tumor/anti-tumor effects. Il-10 binds to the corresponding receptor and initiates transcription of target genes by activating JAK1 and Tyk2, which subsequently leads to phosphorylation of STAT3 (21, 22). Clinically relevant studies have demonstrated that NSCLC patients expressing high levels of IL-10 have poor prognosis (23, 24).However, it has also been reported that insufficient expression of IL-10 in tumors is a negative prognostic factor for early-stage NSCLC (21, 25, 26). These inconsistent studies on IL-10 suggest that the cellular source of IL-10 and the effects of IL-10 on different cell types are what determine the ultimate role of IL-10 in cancer (27).

Finally, we also observed that IL-17 concentrations in lung cancer patients after the second cycle of immunotherapy were negatively associated with CB. Studies have shown that the IL-17 signaling pathway can increase the immunosuppressive activity of regulatory T cells, leading to tumor growth and development (28).High concentrations of baseline serum IL-17 were identified in ipilimumab-treated metastatic melanoma patients developing severe grade 3 gastrointestinal irAEs and may thus serve as a putative biomarker for defining both at-risk patients and the severity of ipilimumab-induced colitis (29).

With close collaborations between academia and industry, recombinant IFNα2 became the first human immunotherapeutic approved by the US Food and Drug Administration (FDA) for cancer and, other than insulin, the first FDA-approved pharmaceutical product produced by recombinant DNA technology (30). IFNα has multiple antitumor properties, including direct tumor cell killing and stimulation of host immune cells, including dendritic cells and CD8+ T cells (31–33). However, no association has been found between the level of IFN-α and immune-related adverse events. According to our results, we can explain why overactivated immune cells can also damage other normal cells, which may lead to immune-related adverse events.

IFN-γ has various roles in immune reactions against tumors, including stimulation of tumor-infiltrating lymphocyte (TIL) proliferation and differentiation and secretion of IFN-γ following activation of T lymphocytes by tumor antigens (34). In contrast, IFN-γ may also promote the production of immunosuppressive molecules, which can have direct negative feedback on effector T cell function (35). During the elimination phase of the immune response against tumor cells, recruited tumor-infiltrating macrophages and NK cells produce various cytokines, including IFN-γ, to kill tumor cells (36). Therefore, an elevated level of IFN-γ may suggest increased cytotoxic activity against lung cancer tumor cells. However, this mechanism of action can also give rise to autoimmune-like side effects known as irAEs. In a study by Constantini (15) IFN-γ levels at nivolumab initiation and two months later did not show correlations with the objective response rate, clinical benefit, or survival, which is consistent with our study.

The types of inflammatory factors produced by different lung cancer patients receiving immune checkpoint inhibitor therapy and the body’s response to the drug treatment, and the activated immune inflammatory pathways are also different. We can further clarify the relationship between cytokine level changes during treatment and the efficacy of immunotherapy by observing the longitudinal cyclical trend of cytokine level changes. In addition, the follow-up period for which clinical data are available is relatively short, and we need to evaluate the significance of these peripheral blood biomarkers in terms of long-term clinical benefit. At the same time, the small sample size may also affect the results of our statistical analysis, which should be addressed in future studies.

In the past decades, cytokines and cytokine receptors have been extensively studied as cancer targets or cancer therapy by enhancing the growth inhibitory and immunostimulatory effects of interferons and interleukins, such as IL-2, IL-7, IL -12 and IL-15, or by inhibiting the inflammatory and tumor-promoting effects of cytokines such as TNF, IL-1β and IL-6 (10). For some cytokines, their ability to initiate pleiotropic immune responses can both increase antitumor immunity and decrease autoimmunity, which may improve their potential for clinical use with immunotherapy, especially in mitigating irAEs. The emergence of immunotherapy and an improved understanding of the tumor microenvironment have provided new approaches for the use of cytokines to treat tumors, including the use of cytokine based therapies to enhance antitumor activity or mitigate immune-related adverse reactions. Many challenges remain, especially due to the pleiotropic and often conflicting roles of many cytokines. The carcinogenic and anticancer mechanisms of cytokines still need to be confirmed by a large number of pre-clinical studies, so their anti-tumor efficacy can only be revealed in the future.

At present, a large number of targeted treatments for irAEs with cytokine antibodies have been reported, suggesting that cytokines are both effector molecules in the anti-cancer effects of immune checkpoint inhibitors and contributors to the mechanism of irAEs development. We found the cytokines as predictive precursors for irAEs. With an increasing number of studies highlight the ability of next-generation immunotherapies to engage individual cytokines in controlling anti-tumor immune responses, more research is needed to determine their impact on irAEs development. Our study showed that IL-1, IL-2, IL-4, IL-5, IL-12, IL-17, IFN-α, IFN-γ, and TNF-α were not associated with the prediction of immunotherapy efficacy, which was related to the relatively short follow-up period for which we could obtain clinical data, and the small sample size may also affect the results of our statistical analysis. This problem should be addressed in future studies.

## Conclusion

Cytokine serum levels may provide prognostic information and constitute predictive markers of immunotherapy benefits in patients with lung cancer. Further studies of the predictive effects of these markers in larger populations are warranted.

## Data availability statement

The raw data supporting the conclusions of this article will be made available by the authors, without undue reservation.

## Ethics statement

The studies involving human participants were reviewed and approved by The First Affiliated Hospital of Xi’an Jiaotong University,2020(G175). Written informed consent for participation was not required for this study in accordance with the national legislation and the institutional requirements.

## Author contributions

Study concept and design: NZ and CL. Acquisition, analysis, or interpretation of data: All authors. Drafting of the manuscript: NZ. Critical revision of the manuscript for important intellectual content: All authors. Statistical analysis: NZ. All authors contributed to the article and approved the submitted version.
