# Corrigendum: Serum cytokine levels for predicting immune-related adverse events and the clinical response in lung cancer treated with immunotherapy

**DOI:** 10.3389/fonc.2022.1069999

**Published:** 2022-12-13

**Authors:** Ni Zhao, Ye Yi, Wen Cao, Xiao Fu, Nan Mei, Chunli Li

**Affiliations:** ^1^ Department of Medical Oncology, The First Affiliated Hospital of Xi’’an Jiaotong University, Xi’an, China; ^2^ Department of Respiratory Medicine, The First Affiliated Hospital of Xi’an Jiaotong University, Xi’an, China; ^3^ Department of Hematology. The First Affiliated Hospital of Xi’an Jiaotong University, Xi’an, China

**Keywords:** biomarkers, cytokines, immunotherapy, lung cancer, immune related adverse events

In the published article, there was an error in the legend for **Figure 12** and **Figure 13** as published. There was a mistake in the legend for **Figure 12** and **Figure 13**, an incorrect description was used.

**Figure 12:**
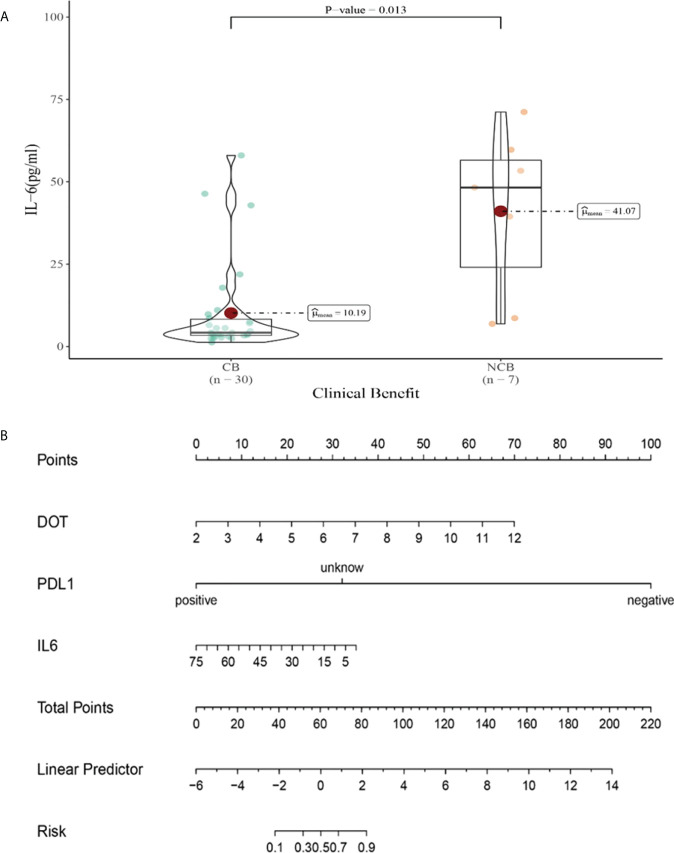
**(A)** Differences in IL-6 levels after the third cycle therapy between CB and UCB; **(B)** The nomogram based on logistic multivariate analysis.

**Figure 13:**
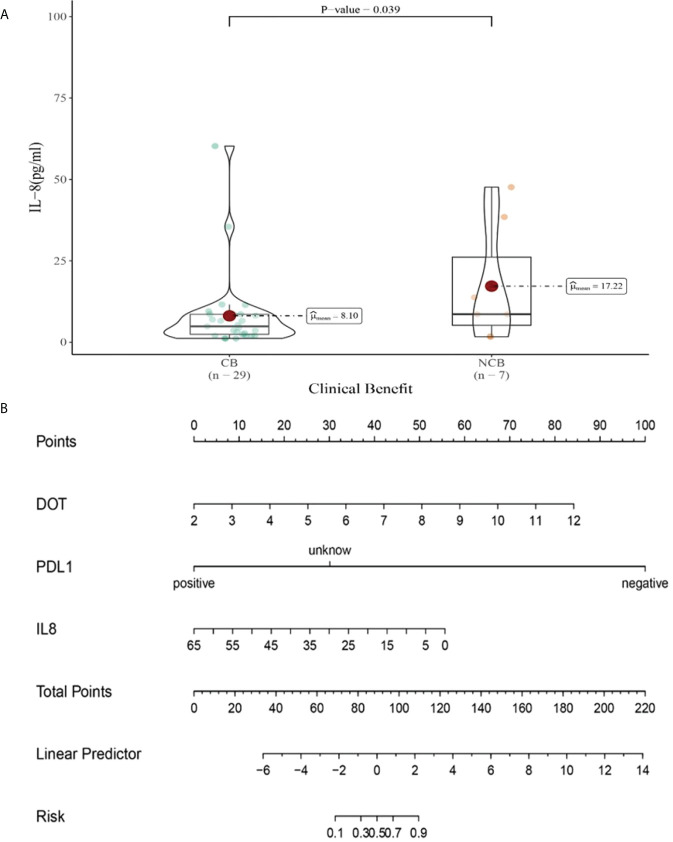
**(A)** Differences in IL-8 levels after the third cycle therapy between CB and UCB; **(B)** The nomogram based on logistic multivariate analysis.

“**Figure 12**. (A) Differences in IL-6 levels after the second cycle therapy between CB and UCB; (B) The nomogram based on logistic multivariate analysis.” should be “**Figure 12**. (A) Differences in IL-6 levels after the third cycle therapy between CB and UCB; (B) The nomogram based on logistic multivariate analysis.”

“**Figure 13** (A) Differences in IL-8 levels after the second cycle therapy between CB and UCB; (B) The nomogram based on logistic multivariate analysis.” should be “**Figure 13** (A) Differences in IL-8 levels after the third cycle therapy between CB and UCB; (B) The nomogram based on logistic multivariate analysis.”

The corrected legend appears below.

The authors apologize for this error and state that this does not change the scientific conclusions of the article in any way. The original article has been updated.

In the published article, there was an error in **Figure 3** as published. “81 patients had not been treated for six months at the end of follow-up and could not be analyzed for effectiveness.” should be “79 patients had not been treated for six months at the end of follow-up and could not be analyzed for effectiveness.”

The corrected **Figure 3** and its caption appear below.

**Figure 3:**
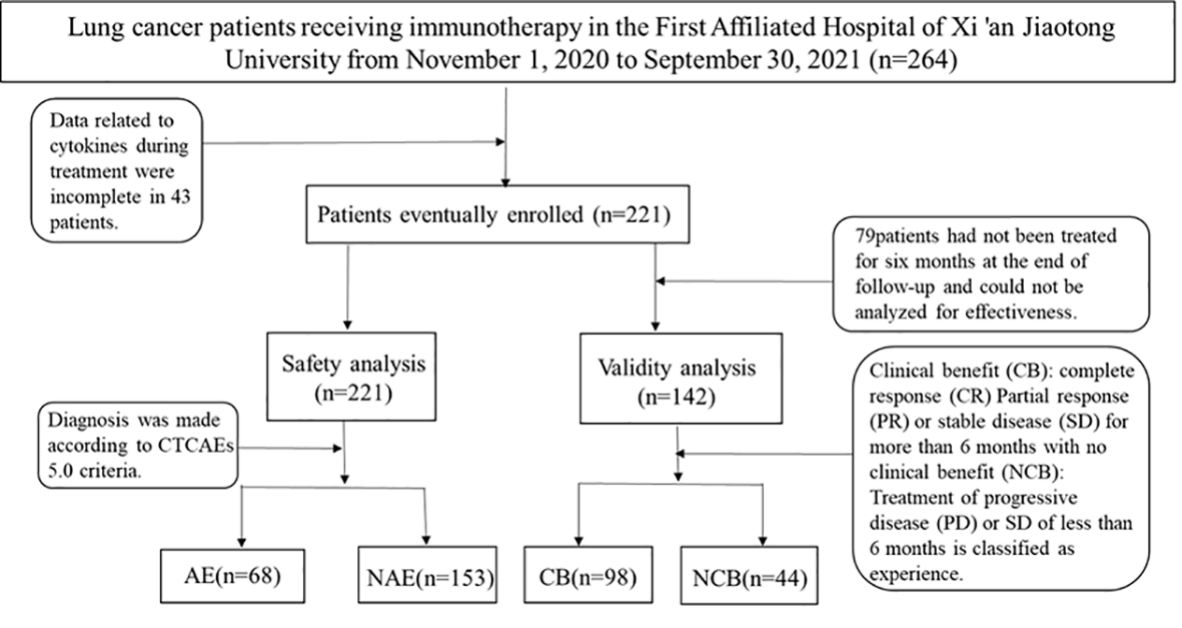
Selection process for patients.

The authors apologize for this error and state that this does not change the scientific conclusions of the article in any way. The original article has been updated.

## Publisher’s note

All claims expressed in this article are solely those of the authors and do not necessarily represent those of their affiliated organizations, or those of the publisher, the editors and the reviewers. Any product that may be evaluated in this article, or claim that may be made by its manufacturer, is not guaranteed or endorsed by the publisher.

